# Safety and efficacy comparison of polyethylene glycol, hemp seed oil, and 5% sugar brine for bowel preparation in older patients: study protocol for a randomized controlled trial

**DOI:** 10.1186/s13063-022-07059-1

**Published:** 2023-03-06

**Authors:** Xing Wang Zhu, Jun Yan, Long Miao, Ying Li He, Hai Ping Wang, Xun Li

**Affiliations:** 1 The First School of Clinical Medicine, Lanzhou University, Lanzhou, 730000 China; 2 Department of General Surgery, The First Hospital of Lanzhou University, Lanzhou, 730000 China; 3 Key Laboratory of Biological Therapy and Regenerative Medicine of Gansu Province, Lanzhou, 730000 China

**Keywords:** Colonoscopy, Bowel preparation, Hemp seed oil, Polyethylene glycol, Boston Bowel Preparation Scale

## Abstract

**Background:**

The incidence of colorectal cancer among the middle-aged and elderly is gradually increasing in China. Colonoscopy is an effective method for the early diagnosis of colorectal cancer, and bowel preparation is one of many important factors affecting colonoscopy. Although there are many studies on intestinal cleansers, the results are not ideal. There is evidence that hemp seed oil has certain potential effects in intestinal cleansing, but prospective studies on this topic are still lacking.

**Methods:**

This is a randomized, double-blind, single-center clinical study. We randomly assigned 690 participants to groups each administered 3 L of polyethylene glycol (PEG), 30 mL of hemp seed oil and 2 L of PEG, or 30 mL of hempseed oil, 2 L of PEG, and 1000 mL of 5% sugar brine. The Boston Bowel Preparation Scale was considered the primary outcome measure. We evaluated the interval between consumption of bowel preparation and first bowel movement. Secondary indicators included the time of cecal intubation, detection rate of polyps and adenomas, willingness to repeat the same bowel preparation, whether the protocol was tolerated, and whether there were adverse reactions during bowel preparation and were evaluated after counting the total number of bowel movements.

**Discussion:**

This study aimed to test the hypothesis that hemp seed oil (30 mL) increases the quality of bowel preparation and reduces the amount of PEG. Previously, we found that its combination with 5% sugar brine can reduce the occurrence of adverse reactions.

**Trial registration:**

Chinese Clinical Trial Registry ChiCTR2200057626. Prospectively registered on March 15, 2022

**Supplementary Information:**

The online version contains supplementary material available at 10.1186/s13063-022-07059-1.

## Administrative information

**Table:** 

Title	Safety and efficacy comparison of polyethylene glycol, hemp seed oil, and 5% sugar brine for bowel preparation in older patients: study protocol for a randomized controlled trial
Trial registration	Chinese Clinical Trial Registry ID: ChiCTR2200057626. Registered on March 15, 2022
Protocol version	December 31, 2021, version 1.0
Funding	This study received research grants from (1) Gansu Provincial Key Talent Project (grant number: 9 of 2020); (2) Science Foundation of the First Hospital of Lanzhou University: ldyyyn2021-108 and ldyyyn2021-102; (3) Gansu Provincial Higher Education Industry Support Program: 2022CYZC-05.
Author details	XingWang Zhu^1#^, Jun Yan^2,3#^, Long Miao^2^, YingLi He^2^, HaiPing Wang^2,3^, Xun Li^1,2,3*^ ^1^The First School of Clinical Medicine, Lanzhou University ^2^Department of General Surgery, The First Hospital of Lanzhou University ^3^Key Laboratory of Biological Therapy and Regenerative Medicine of Gansu Province, The First School of Clinical Medicine, Lanzhou University
Name and contact information for the trial sponsor	Email: lxdr21@126.com
Role of sponsor	The sponsor has no role in the design of the study, collection, analysis, and interpretation of the data or writing of the manuscript.

## Introduction

Colorectal cancer is a common malignant tumor of the digestive system. According to a report of the cancer survey, colorectal cancer ranks in the top three in new cases and deaths in both men and women, making it one of the most life-threatening cancers among Chinese patients [1, 2]. The progression of colorectal adenomas to cancer may take up to 10 years, and the incidence increases with age [3–6]. Moreover, the risk of mortality from colorectal cancer has been reported to decrease by 53% in patients who underwent endoscopic adenoma resection [7–10]. Many factors affect the quality of colonoscopy, of which bowel preparation is an essential factor. Inadequate bowel preparation can reduce the adenoma detection rate, prolong the duration of colonoscopy, and increase the risk of examination [11–13].

Currently, polyethylene glycol (PEG) is still the first choice for clinical intestinal preparation. Due to its poor taste, the need to drink a lot of water and adverse events, such as nausea and vomiting, patients have reduced tolerance and medical compliance for PEG [14, 15]. According to a survey, the incidence of constipation is higher among middle-aged and elderly patients than among younger patients owing to factors such as a decline in intestinal function and dietary habits, affecting the quality of bowel preparation [16, 17]. Recently, many clinical studies on bowel preparation are being conducted with the aim of improving the quality of bowel preparation and patients’ tolerance. Examples of such studies include those on the application of ascorbic acid, senna, and sodium phosphatases [18–21]. However, many trials have not achieved the expected results pertaining to bowel preparation. Therefore, improving the quality of bowel preparation and reducing the amount of PEG is still a problem that needs to be solved.

Hemp seed has been used as food and medicine in China for more than 3000 years, and it is listed as a Chinese medicinal material of the same origin as medicine and food by the National Health Commission [22, 23]. It can regulate constipation, improve immunity, and treat diseases of the gastrointestinal tract. There are many mechanisms through which hemp seed regulates constipation, including regulating intestinal flora, promoting the growth of probiotics to improve intestinal function, and regulating ion channels on the surface of intestinal epithelial cells, which play a role in regulating constipation [24–26]. Currently, there are many clinical studies on Maziren pills formulated with hemp seed, which have found that it has a good curative effect on constipation in elderly patients [27–29]. Hemp seed oil is a highly nutritious vegetable oil obtained by pressing or leaching hemp seed. It is rich in unsaturated fatty acids, amino acids, vitamin E, alkaloids, and other components with high nutritional value [30]. The unsaturated fatty acids present in hemp seed help regulate blood lipids and the immune system [31, 32]. Despite hemp seed oil having high edible and medicinal value, it contains delta-9-tetrahydrocannabinol acid (Δ9-THCA). At certain concentrations in the body, it may produce symptoms such as abdominal distension, sweating, and vomiting, which are the main adverse reactions [33–35]. According to the Chinese dietary nutrition guidelines, the recommended daily intake of the edible oil is 25–30 g. This study used 30 mL of hemp seed oil that meets the edible standard as an adjuvant drug for bowel preparation.

Hemp seed oil has a laxative effect; thus, it was combined with PEG to reduce the amount of PEG used in this study. Moreover, due to the poor taste of PEG and some adverse events caused by insufficient caloric intake during intestinal preparation, patients’ bowel preparation can be poor. Therefore, 5% sugar brine (5% glucose and 0.9% sodium chloride) was added to improve the taste of PEG and replenish energy and electrolytes. Additionally, 5% sugar brine is isotonic, can prevent dehydration caused by catharsis, and keep the intestinal mucosal cells in an isotonic state. When the fluid flow in the intestine exceeds a certain flow rate, the emptying of intestinal contents can be accelerated, which can assist in intestinal cleansing.

Therefore, we hypothesized that the oral administration of 30 mL hemp seed oil in stages could enhance the intestinal cleansing ability of PEG, decrease the dosage of PEG without any obvious adverse effects, and increase its acceptance. This trial aimed to determine the efficacy, safety, and tolerability of 2 L of PEG plus hemp seed oil in combination with 5% sugar brine for bowel preparation.

## Methods

This protocol was developed according to the Recommendations for Interventional Trials (SPIRIT) [36]. The trial was registered at the Chinese Clinical Trial Registry (www.chictr.org.cn) with the identifier ChiCTR2200057626 and approved by the local ethics committee (approval number: LDYYLL2022-55). Written informed consent was obtained from all enrolled patients.

### Objectives

This study aimed to investigate the potential of 30 mL of hemp seed oil in enhancing bowel cleansing. The study also evaluated the effect of reducing PEG by half by adding hemp seed oil and 5% sugar brine on reducing the adverse effects of bowel preparation and increasing the patients’ tolerance of PEG.

### Trial design and setting

**Fig. 1:**
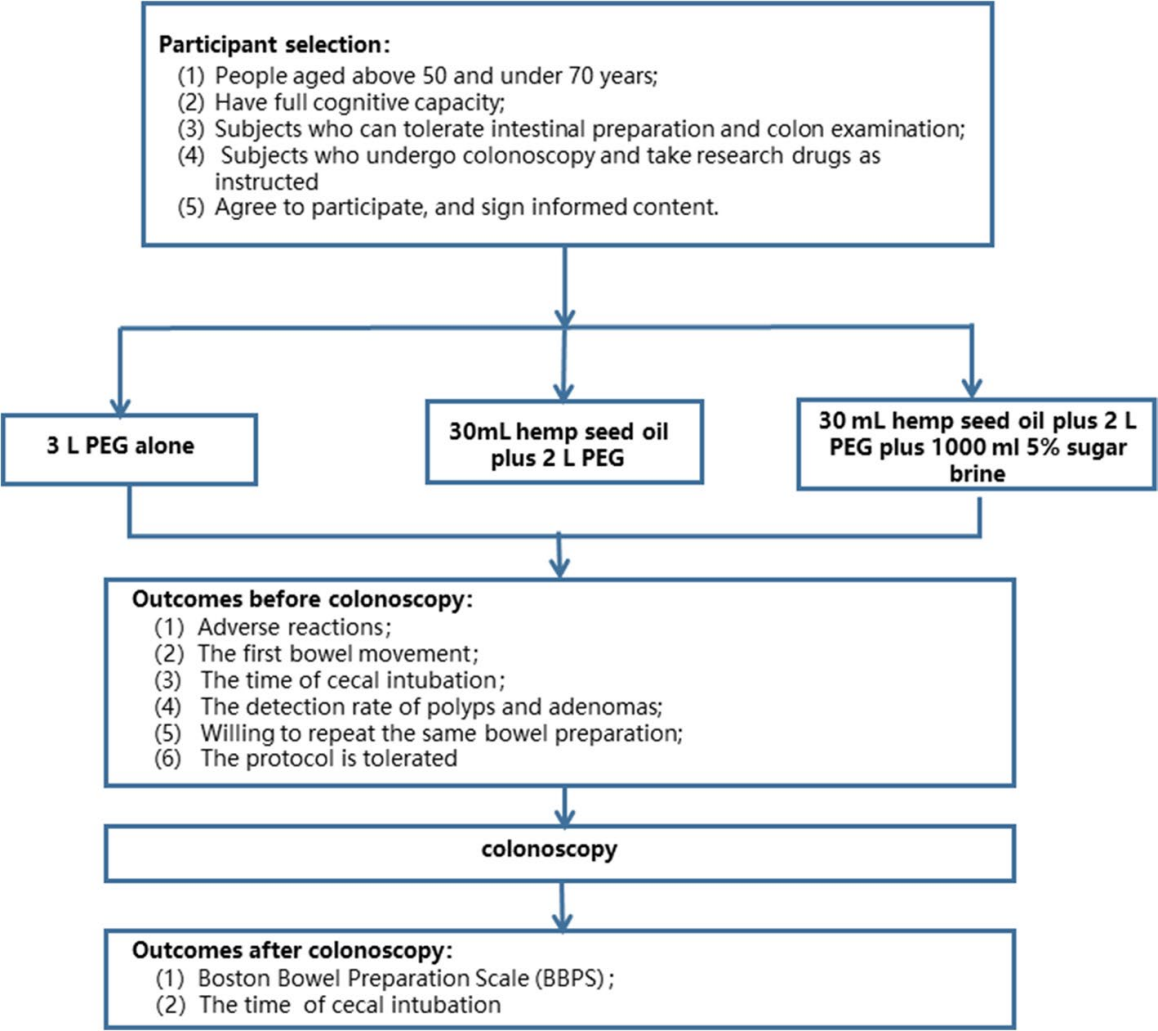
Flow diagram of the patient’s enrolment. PEG, polyethylene glycol

This is a double-blind, single-center, three-arm, non-inferiority study that will be conducted at the Surgical Endoscopy Center of the First Hospital of Lanzhou University from April 2022 to February 2023. Recruited participants will be randomly assigned to receive a combination of 3 L of PEG, 30 mL of hemp seed oil and 2 L of PEG, or 30 mL of hemp seed oil, 2 L of PEG, and 1000 mL of 5% sugar brine (Fig. 1).

### Participant selection

The following are the inclusion criteria: (1) age 50–70 years, (2) full cognitive capacity, (3) tolerance of intestinal preparation and colon examination, (4) patients undergoing colonoscopy, and (5) patients who agreed to participate, take research drugs as instructed, and sign informed content.

The following are the exclusion criteria: (1) participants who are allergic to any component of the experimental drug; (2) participants who cannot tolerate ordinary colonoscopy; (3) participants who have used research drugs, other intestinal preparations, or drugs that affect gastrointestinal motility within 7 days from the start of the trial; (4) participants who are diagnosed or suspected to have gastrointestinal obstruction, gastric retention, gastroparesis, disturbance of gastric emptying, or acute gastrointestinal bleeding; (5) participants with suspected abdominal organ perforation, including gastric perforation, intestinal perforation, and appendix perforation; (6) participants with a history of major gastrointestinal surgery; (7) women with positive pregnancy tests or pregnancy plans during the screening period; (8) participants with neurological diseases; (9) patients with severe heart disease or electrolyte imbalance that is difficult to correct; and (10) participants in other clinical trials within the last 3 months.

### Sample size

We calculated the expected sample size based on the preliminary extrapolation of the results of bowel preparation before the study began. According to the results of previous studies, when using 3 L PEG for bowel preparation, the qualified rate of bowel preparation was 80%. PEG was accepted as the standard preparation, and the aforementioned percentage was taken as a reference [18, 20, 37, 38]. Assuming the qualified rate of bowel preparation in this study is 85%, the alpha level was set to 0.05. When a sample size of 690 was calculated, a 5% difference between the three groups could be determined.

### Recruitment, randomization, and blinding

Investigators trained for the study and authorized by the principal investigator will assess the candidates according to the inclusion and exclusion criteria on the day before the colonoscopy. After eligible participants are identified, written informed consent will be obtained from each eligible patient, next of kin, or legal representative. On the consent form, participants will be asked whether they agree to the use of their data and biological specimens. Participants will be asked to agree to the research team sharing relevant data with regulatory authorities as appropriate. Researchers should collect clinical data, including gender, age, weight, BMI, history of the previous colonoscopy, history of abdominal surgery, cardiovascular and neurological diseases, and long-term use of certain drugs.

Initially, the SPSS version 22.0 software will be used to generate random sequences, and these will be subsequently put in an opaque envelope. The independent statistical expert used a computer to generate random numbers, and the participants were assigned to the following 3 groups in a 1:1:1 ratio, numbered sequentially after random assignment: A, 3L PEG; B, 30 mL of hempseed oil and 2 L of PEG; and C, 30 mL of hemp seed oil, 2 L of PEG, and 5% sugar brine. Moreover, researchers, endoscopic physicians, and participants will all be blinded during the examination to eliminate bias as much as possible.

At the end of the study, unblinding will be carried out by the principal investigator. The first unblinding will be conducted to uncover the groups of participants, followed by statistical analysis to clarify the differences between the groups, and a second unblinding will be conducted to further clarify the specific groups. When an emergency, such as a serious adverse event, threatens the safety of a subject and the subject’s grouping must be known, an emergency unblinding would be conducted. The unblinding of the specific time, reason, and name of the executive of the emergency will be timely recorded.

### Study protocol

All subjects undergoing colonoscopy should start a no-pigment, low-residue, low-fiber diet 3 days before examination and fast at 18:00 the night before the examination. If hypoglycemia or other discomforts occur due to hunger on the same day, oral colorless vitamin drinks or candy bars can be taken to enhance tolerance. Moreover, to allay the participants’ fear of intestinal preparation, researchers should explain the purpose of colonoscopy, importance of adequate intestinal preparation, and potential adverse events that may occur during intestinal preparation.

**Fig. 2:**
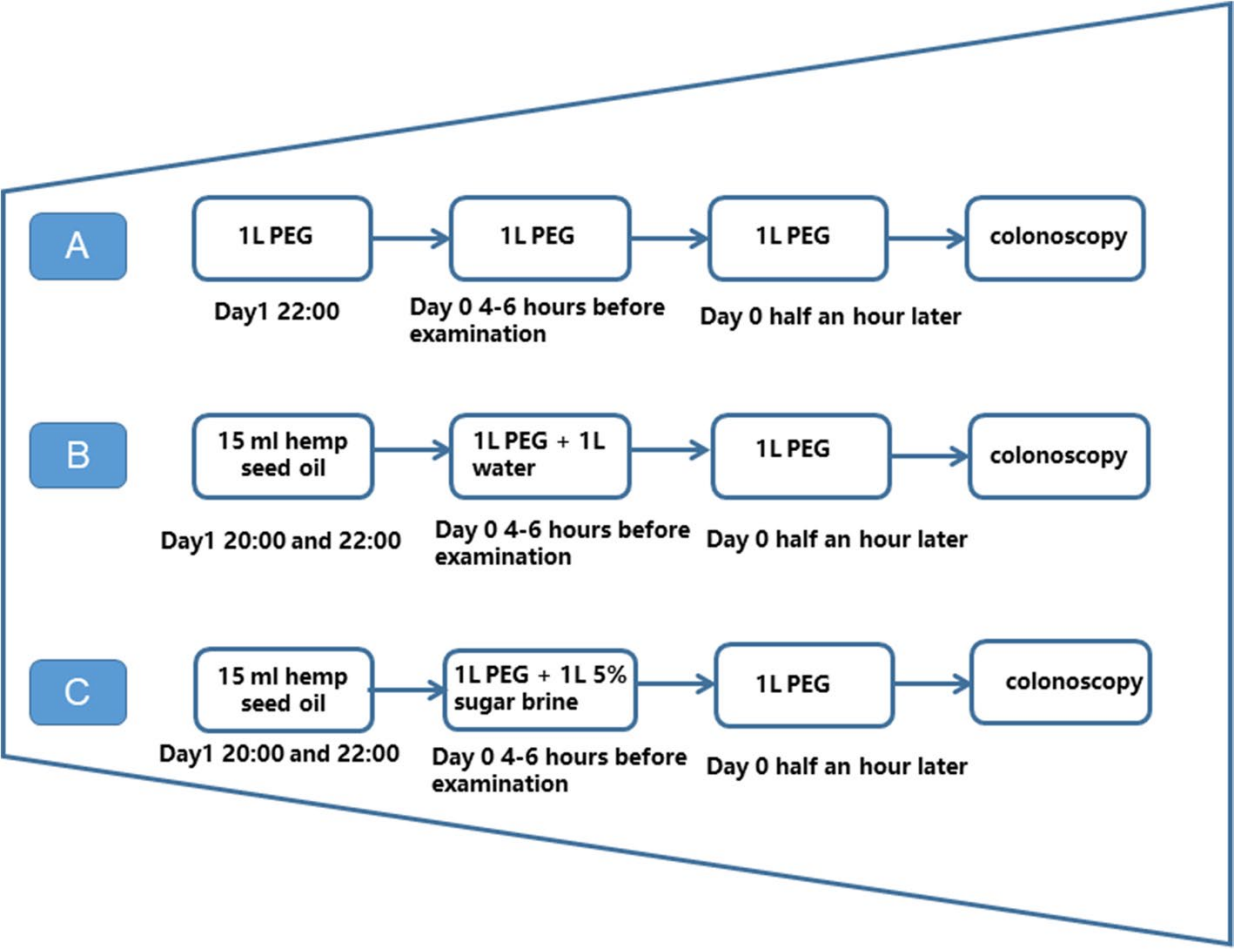
Study protocol for each group. **A** 3 L PEG. **B** 30 mL of hempseed oil and 2 L of PEG. **C** 30 mL of hempseed oil, 2 L of PEG, and 5% sugar brine

**Fig. 3:**
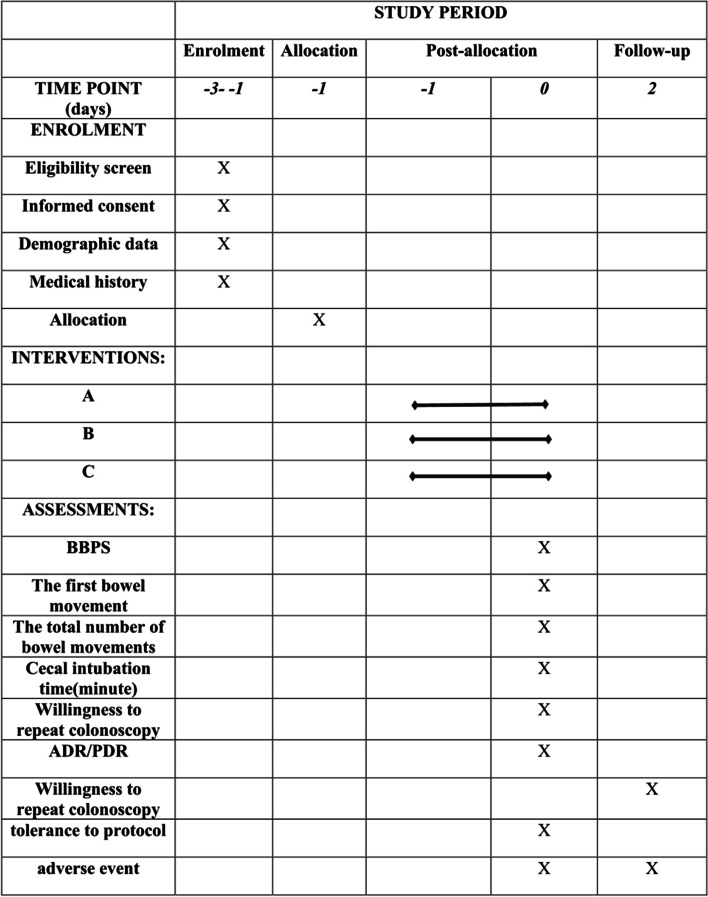
Study schedule of enrollment, interventions, and assessments. **A** 3 L of PEG. **B** 30 mL of hempseed oil and 2 L of PEG. **C** 30 mL of hempseed oil, 2 L of PEG, and 5% sugar brine. BBPS, Boston Bowel Preparation Scale; ADR, adenoma detection rate; PDR, polyp detection rate

Figure 2 depicts the drug use schemes of the 3 groups. The oral intestinal preparation process under the guidance of researchers in the 3 L of PEG group was as follows: one bag of PEG and 1 L of clear fluids was taken orally at 22:00 the day before the examination, the second bag was taken 4–6 h before the examination, and the third bag of PEG was taken half an hour later (Fig. 2A). As for the 30 mL of hemp seed oil and 2 L of the PEG group, patients orally took 15 mL of hemp seed oil in divided doses at 20:00 and 22:00 1 day before the examination, the first bag of PEG and 1 L of clear fluids 4–6 h before the examination, and the second bag of PEG half an hour later (Fig. 2B). Regarding the 30 mL of hemp seed oil, 2 L of PEG, and 5% sugar brine group, patients orally took 15 mL of hemp seed oil in divided doses at 20:00 and 22:00 1 day before the examination, the first bag of PEG and 1000 mL of 5% sugar brine 4–6 h before the examination, and another bag of PEG half an hour later (Fig. 2C). In the study, participants were free to drink clear fluids after the commencement of bowel preparation. The study schedule is shown in Fig. 3.

### Study endpoints

#### Primary outcomes

The effect of intestinal preparation was regarded as the main outcome indicator and was scored using the Boston Bowel Preparation Scale (BBPS). As a commonly used clinical bowel preparation assessment scale, the BBPS score divides the large intestine into 3 segments (rectum and sigmoid colon, transverse colon and descending colon, ascending colon and cecum) and scores after adequate bowel preparation. Each intestinal segment was scored from 0 to 3 points, and the total score ranges from 0 to 9 points. The indications of scorings were as follows: 0 points, the entire intestinal mucosa cannot be observed due to irremovable solid or liquid feces; 1 point, parts of the intestinal mucosa cannot be observed due to the presence of stains, turbid liquid, and residual feces; 2 points, the intestinal mucosa is well observed, but a small amount of stains, cloudy liquid, and feces remain; and 3 points, the intestinal mucosa is well observed, and there are no residual stains, cloudy liquids, and stools [39]. In this study, the total score of BBPS score is > 5, and any intestinal segment ≥ 2 points can be considered satisfactory intestinal preparation.

#### Secondary outcomes

Adverse reactions that occur during bowel preparation are the secondary outcome indicators of the study. We evaluated the interval between taking bowel preparation and the first bowel movement, the total number of bowel movements completed, time of cecal intubation, detection rate of polyps and adenomas, willingness to repeat the same bowel preparation, whether the protocol is tolerated (0 points for no discomfort; 1 point for mild discomfort; 2 points for ability to tolerate; 3 points for not being able to tolerate), and adverse events.

### Rescue therapy

For participants with poor bowel preparation, the endoscopist will determine whether to continue colonoscopy. If colonoscopy cannot be performed because of insufficient bowel preparation, bowel preparation can be performed again free of charge. Moreover, in case of serious adverse reactions, the investigator will provide prompt and appropriate treatment based on the diagnosis and documents related to all treatment options.

### Safety assessments

Responses of all events related to adverse reactions must be recorded, including the duration of symptoms or signs and outcomes. All adverse events related to bowel preparation and colonoscopy in a case report form (CRF), including nausea, vomiting, bloating, abdominal pain, dizziness, vertigo, sweating, dry mouth, and electrolyte imbalance, must be recorded.

### Data management

To promote participant retention, we recruited participants through health promotion and followed up with them by telephone and questionnaires after colonoscopy. All data were recorded in CRF, which contained all data required for the study. Before formally using the data, relevant persons should be trained by experts. Only the researcher or relevant researchers are authorized to use EXCEL 2019 to collect, classify, and use the data. Data collection and analysis are supervised by the Ethics Committee of the First Hospital of Lanzhou University.

All researchers will follow professional confidentiality rules and must keep all personal and medical information of patients confidential. The paper clinical report form will be destroyed 3 years after the completion of the study. Personal patient information will be hidden, electronic data will be stored and encrypted, and access to databases will be restricted. All data management should be conducted by a designated data management team established by the principal investigator and an independent statistician.

### Protocol amendments

The study protocol has been approved by the Ethics Committee of the First Hospital of Lanzhou University. The protocol amendments will be submitted to the Ethics Committee for review, and the online clinical trial registry will be updated.

### Statistical analysis

In this study, we will adopt intent-to-treat and per-protocol methods to analyze all data. The basic information of patients is expressed by mean, median, and standard deviation. The chi-square test will be used to compare the categorical variables in the basic data of patients. In two independent samples, the Mann–Whitney *U*-test and Student’s test will be used for the comparison of continuous variables; the chi-square test will be used for the comparison of the main results; secondary outcomes will be compared using the chi-square test for categorical variables and Student’s *t*-test for continuous variables. All statistical analyses will be conducted with the 2-tailed test, and *P* < 0.05 will be considered statistically significant. We will make every attempt to collect complete information for all participants and avoid missing data. If necessary, missing data will be handled using multiple imputation. As no formal stopping rules have been specified for this study, no formal interim analyses are planned, and hence, no statistical testing will take place until the final analysis. All data were analyzed using SPSS 26 (SPSS Inc., an IBM Company, Chicago, IL).

### Patient and Public Involvement

No patient or public was involved in the design or in the recruitment to and conduct of the study. Upon completion of the study, the results will be disseminated to participants as required.

### Ethics and dissemination

The Ethics Committee of the First Hospital of Lanzhou University approved this study (approval number: LDYYLL 2022-55). Each patient voluntarily signed the informed consent form before they were enrolled in the study. The study will be conducted according to the latest published protocol and the latest version of the Declaration of Helsinki. The final trial process and results will be published in peer-reviewed journals and academic conferences.

## Discussion

Colonoscopy can be used to visually diagnose intestinal diseases and reduce the incidence of colorectal cancer. The survey showed that the incidence of colorectal cancer in China increases with age [40]. Poor bowel preparation is an important factor that affects the failure of cecal intubation during colonoscopy and leads to a missed diagnosis of some polyps or lesions, which may warrant repeated colonoscopy [41]. There are many studies on modified bowel preparations; however, because of the existence of many adverse reactions or poor medical compliance and tolerance by patients, it is necessary to find safer and more effective bowel preparation methods [42–44]. Currently, many studies have demonstrated the laxative effect of hemp seed oil. However, there is no research on its use in bowel preparation, making it necessary to explore whether 30 mL of hemp seed oil can improve the bowel cleansing potential of PEG in bowel preparation.

We designed a randomized, double-blind, single-center clinical trial to investigate whether 30 mL of hemp seed oil can improve the potential of 2 L PEG for bowel preparation and whether a combination of 30 mL hemp seed oil and 5% sugar brine can reduce the required volume of PEG, as well as reduce the adverse reactions during bowel preparation.

The results of this trial will influence evidence-based decision-making for bowel preparation regimen prescriptions as it will be fundamental in providing reliable recommendations for bowel preparation regimens before colonoscopy.

## Trial status

December 31, 2021, protocol version 1.0 is being used. Recruitment began in April 2022 and is estimated to be completed in February 2023.

## Supplementary Information

**Additional file 1: **SPIRIT Checklist for Trials.

## Data Availability

The datasets used and/or analyzed during the current study are available from the corresponding author upon reasonable request.

## Abbreviations

PEG: Polyethylene glycol
BBPS: Boston Bowel Preparation Scale
ADR: Adenoma detection rate
PDR: Polyp detection rate
Δ9-THCA: Delta-9-tetrahydrocannabinol acid
SPIRIT: Recommendations for Interventional Trials
CRF: Case report form
ITT: Intent-to-treat
PP: Per-protocol
